# Plasmid-Mediated Colistin Resistance in *Salmonella enterica*: A Review

**DOI:** 10.3390/microorganisms7020055

**Published:** 2019-02-19

**Authors:** Tiago Lima, Sara Domingues, Gabriela Jorge Da Silva

**Affiliations:** 1 Faculty of Pharmacy, University of Coimbra, 3000-458 Coimbra, Portugal; tiagoventura_@hotmail.com (T.L.); saradomingues@ff.uc.pt (S.D.); 2 Centre for Neuroscience and Cell Biology, University of Coimbra, 3004-517 Coimbra, Portugal

**Keywords:** antimicrobial resistance, colistin, *mcr*, horizontal gene transfer, food safety, epidemiology

## Abstract

Colistin is widely used in food-animal production. *Salmonella enterica* is a zoonotic pathogen, which can pass from animal to human microbiota through the consumption of contaminated food, and cause disease, often severe, especially in young children, elderly and immunocompromised individuals. Recently, plasmid-mediated colistin resistance was recognised; *mcr*-like genes are being identified worldwide. Colistin is not an antibiotic used to treat *Salmonella* infections, but has been increasingly used as one of the last treatment options for carbapenem resistant Enterobacteria in human infections. The finding of mobilizable *mcr*-like genes became a global concern due to the possibility of horizontal transfer of the plasmid that often carry resistance determinants to beta-lactams and/or quinolones. An understanding of the origin and dissemination of *mcr*-like genes in zoonotic pathogens such as *S. enterica* will facilitate the management of colistin use and target interventions to prevent further spread. The main objective of this review was to collect epidemiological data about mobilized colistin resistance in *S. enterica*, describing the *mcr* variants, identified serovars, origin of the isolate, country and other resistance genes located in the same genetic platform.

## 1. Introduction

The overuse and inappropriate use of antibiotics in diverse settings, such as human and veterinary therapeutics, animal production and agriculture, is widely accepted as one of the major causes of the emergence of antimicrobial resistance worldwide [1,2]. During the past decades, we have witnessed the evolution of bacteria by the selective pressure of antibiotics, with new resistance mechanisms and their spread across bacteria populations from various ecological niches. The antimicrobial resistance was responsible for about 700,000 deaths in 2016 and this number is estimated to increase to 10 million annual deaths by 2050 [2].

In human medicine, the treatment of infections due to multidrug resistant bacteria is a real challenge, like those caused by *Pseudomonas aeruginosa*, *Acinetobacter baumannii* and carbapenem-resistant Enterobacteria. The void of effective antibiotics led to the recent use of an old antibiotic, colistin, as one of the last-resort therapeutic options. The World Health Organization reclassified colistin as an antibiotic of critical importance in human clinical settings [3].

However, colistin has been widely used in animal production in several countries for therapeutic, prophylactic and growth promotion purposes [4,5]. The use of low-dose and prolonged course of antibiotics in livestock is clearly associate with selection of zoonotic resistant strains that can be spread by direct contact of animal-to-human or indirectly, like by the food chain [6,7]. The dissemination of resistance determinants is fueled by lateral gene transfer mechanisms, such as conjugation [8]. Animal gut colonizers can exchange genetic material with other bacteria, commensal or pathogenic. Until 2015, known colistin resistance mechanisms were all chromosomally encoded. However, a colistin-mediated resistance gene (*mcr-1* gene) was further identified in a conjugative plasmid in *Escherichia coli* isolates of animal origin from China [9], which generated a wave of concern over the scientific community. Since then, numerous studies have reported plasmid-borne *mcr* alleles, mostly in *E. coli* of animal origin [10,11,12,13,14].

*Salmonella enterica* is an important zoonotic pathogen both in developing and industrialized countries, which can colonize the adult animals gut, especially in poultry and swine [7]. The *mcr* genes have also been found in *S. enterica*, though more infrequently than in *E. coli*, including in *S. enterica* serovar Paratyphi (from now on designated as *S*. Paratyphi) [15], a serotype associated to the development of human enteric fever. This communication summarizes the studies on the epidemiology of plasmid-mediated colistin resistance in *S. enterica*, considering the relevance of *Salmonella* serovars identification, geographic location of isolation and multidrug resistance profile. 

## 2. Colistin Use: Past and Present

Colistin is a polypetide antibiotic that belongs to the class of polymyxins, produced by *Paenibacillus polymyxa*. This class is one of the primary classes of antibiotics with activity against most Gram-negative bacteria and consists of polymyxins A, B, C, D and E, of which only colistin (polymyxin E) and polymyxin B are used in clinical practice [5]. After its discovery in 1947, colistin was used in human medicine in Japan and Europe, but in the 1970s their use was reconsidered due to its neurotoxicity and nephrotoxicity. However, colistin has been widely used in veterinary medicine for the treatment and prevention of infectious diseases in Asian, European and North American countries [9,16,17,18]. Colistin has also been used in the livestock and seafood industry to promote animal growth [19]. 

In the past decade, the global emergence of carbapenemase-producing *Enterobacteriaceae* led to the re-use of colistin administration as a last therapeutic option for treating human infections, with the inevitable risk of emerging resistance [9,20]. The initial target of colistin is lipid A, a component of the lipopolysaccharide (LPS) located in the Gram-negative bacteria outer-membrane (OM), which plays an essential role in cell permeability. The electrostatic interaction between the positively-charged diaminobutyric acid (Dab) residues of colistin and the negatively-charged phosphate groups of lipid A leads to the displacement of divalent cations Ca^2+^ and Mg^2+^, which destabilize the molecule and triggers the permeability of OM, facilitating the entry of colistin by a self-promoted uptake mechanism. Colistin is bactericidal and its action results in leakage of citoplasmic content and cell death [21,22].

## 3. Resistance to Colistin

Colistin resistance is mainly associated with LPS modifications, with consequent reduced or absent affinity to colistin; the underlying mechanism, although common in Gram-negative bacteria, may differ between species [23,24]. It is the lipid A moiety of LPS that suffer changes, essentially due to addition of 4-amino-4-deoxy-l-arabinose (l-Ara4N) and/or phosphoethanolamine (PEtn). These molecules, positively charged, reduce the overall negative charge of LPS and, consequently, of the OM leaflet of the bacterial cells, leading to a smaller electrostatic interaction with the positive charges of colistin, preventing cell lysis [4,23].

Plasmid-mediated colistin resistance is conferred by *mcr* genes, which encode a phosphoethanolamine transferase that add PEtn to lipid A, contributing, like in chromosomal resistance, to decreased binding of colistin to LPS [4,10].

The *mcr-1* gene was identified for the first time in an IncI_2_ plasmid named pHNSHP45. After this first detection, *mcr-1* and its very similar genetic variants were widely identified in diverse *Enterobacteriaceae* of different origins. Nowadays, this gene has been found in approximately 40 countries across five different continents [10,12,25]. This ubiquitous dissemination of the *mcr-1* gene suggests that the use of colistin has probably accelerated the dissemination of *mcr-1* gene in animals and humans [10]. Moreover, several other *mcr* homologs were subsequently identified in *E. coli* and other Gram-negative bacteria. Currently, eight types of *mcr* genes (*mcr-1* to *-8*) have been described and deposited into GenBank. The first reported variants were isolated from animals in Europe and China. The *mcr-2* gene was found for the first time in *E. coli* from pigs and calves in Belgium [26], *mcr-3* in *E. coli* from pigs in China [13], *mcr-4* in a strain of the monophasic variant *S. enterica* serovar Typhimurium from pigs in Italy [14], *mcr-5* in *S*. Paratyphi B dTa+ from poultry in Germany [15], *mcr-6* (previously named *mcr-2.2*) in *Moraxella* spp. isolated from pigs in Great Britain [27], *mcr-7* in three isolates of *Klebsiella pneumoniae* from chickens in China [28] and finally *mcr-8* in NDM-producing *K. pneumoniae* isolates from both pigs and humans in China [25].

All these findings suggest that animals are the reservoir of the *mcr* genes with emphasis on the pigs, mostly due to the heavy usage of polymyxins in food animal production for therapy, prophylaxis and metaphylaxis purposes, which contributes for selection of *mcr* producers. Furthermore, the reports of identification of *mcr* genes have been mostly from animal isolates when compared with human isolates, sustaining animals as the main reservoir. Moreover, some genetic elements, like other resistance genes, insertions sequences and plasmids that are more prevalent and widespread in bacteria of animal origin, are found closely associated with the *mcr*-like genes [29].

## 4. *Salmonella enterica*: Salmonellosis and Enteric Fever in Humans

*S. enterica* infections are an important public health concern worldwide. *S. enterica* serovars can be separated in two main groups: The typhoidal *Salmonella* that comprise *S. enterica* serovar Typhi (from now on designated as *S*. Typhi), *S*. Paratyphi A, *S*. Paratyphi B, and *S*. Paratyphi C, whereas all the other serovars are called as non-typhoidal *Salmonella* (NTS) [30].

Animals are the primary reservoir of NTS, and NTS infections, generally called salmonellosis, are a huge threat in developing countries especially in infants, young children and in HIV-carriers, while in developed countries infection is mostly acquired through the food chain by ingestion of commercially contaminated produced animal-derived food [7,31,32]. It is estimated that NTS gastroenteritis is responsible for about 93.8 million illness and 155,000 deaths each year worldwide, and of these, it is estimated that 80.3 million cases are foodborne, with very high associated costs, most of them in developing countries, which contrasts with the reality in developed countries, where this rate is lower [33]. 

Despite food producing animals behave as the main reservoirs of *S. enterica*, a small group of serovars are capable of infecting and colonizing only determined hosts. For example, typhoidal serovars are human host-restricted organisms that cause typhoidal fever and para-typhoid fever (both also known as enteric fever) [30,34].

All typhoidal *Salmonella* serovars are responsible for 27 million annual cases of enteric fever, which results in more than 200,000 deaths worldwide [35]. In developing countries, where sanitary conditions and clean water are a problem of public health, enteric fever is generally endemic. Fecal-oral route is the main cause for spread of typhoidal *Salmonella*. In some countries, especially in Southeast Asia, *S*. Paratyphi infections are increasing. It is estimated that this serovar is responsible for about half of all enteric fever cases [36].

Currently, colistin is not used to treat human infections caused by this bacterium, and the development of colistin resistance is clinically not relevant. However, in vivo colistin resistance has been observed in *S. enterica* from food-producing animals [37,38,39,40], and the resistance determinants when inserted in genetic mobile elements (e.g., *mcr*-like genes) can be laterally transferred to other species, commensals or pathogens of animal and human origin. Moreover, the genetic platforms carrying *mcr*-like genes frequently host resistance genes that hinder the efficacy of other antibiotic classes [41]. Therefore, the presence of *mcr*-like genes should not be neglected in this zoonotic pathogen.

## 5. Colistin Resistance in *Salmonella enterica*

*S. enterica* strains have developed resistance to a variety of antimicrobials. Chloramphenicol was the first antibiotic used in the treatment of typhoid fever, but emergence of resistance soon after its introduction lead to the replacement by trimethoprim-sulfamethoxazole and ampicillin or amoxicillin. Multidrug resistant strains emerged with the overuse of these first-line treatment drugs, and fluoroquinolones, such as ciprofloxacin, and extended-spectrum cephalosporins, such as ceftriaxone, were introduced in the treatment of *Salmonella* infections. However, resistance to these antimicrobials is now also frequent [7,30,42].

In *S. enterica*, the chromosomal colistin resistance involve activation of the PmrA/PmrB and PhoP/PhoQ two-component regulatory systems, which are responsible for the biosynthesis of L-Ara4N and PEtn. The activation of these systems is related with environmental stimuli, such as low concentration of Mg^2+^, or with specific mutations in the two-component regulatory systems-encoding genes [4,23,43]. These mutations lead to the constitutive expression of PmrA/PmrB and PhoP/PhoQ, with consequent activation of operons *arnBCADTEF* and *pmrCAB,* and permanent addition of L-Ara4N and PEtn, respectively, to lipid A [23].

Other alterations, such as deacylation of lipid A by PagL [23,44], and activation of the transcription of genes involved in adaptation and survival of the bacterial cells by RpoN [23,45], can also lead to colistin resistance in *S. enterica*, but are less common.

Plasmid-mediated colistin resistance conferred by *mcr-1* [46], *mcr-2* [47], *mcr-3* [48], *mcr-4* [14] and *mcr-5* [15] genes have been already identified in different serovars of *S. enterica*. Like in other bacterial species, *mcr*-like genes have been detected in isolates from different origin, such as food-producing animals, food products and human samples, and are inserted in diverse genetic environments and plasmid backbones. It is of note that the presence of the *mcr* genes can be associated with low level of resistance to colistin [4,14,15,46,49,50,51], allowing to persist undetectable.

Table 1 summarizes the reports on *mcr*-like genes and their variants in this species and the key findings of each study. Briefly, *S*. Typhimurium is the most prevalent serotype harbouring *mcr* genes. This serotype is also one of the most frequent to cause human infections [52]. Monophasic variants of *S*. Typhimurium such as 1,4,[5],12:i:- are also widely reported. It is still worth noting that *mcr* positive Paratyphi B are isolated from animal samples, though this serotype usually infects humans and cause invasive disease [52]. Food-producing animals appear to be the main reservoir of *mcr* positive *S. enterica* strains. Poultry and swine animals are the most reported sources of isolates. Nonetheless, there are isolates from human clinical sources, which suggests dissemination from animals to humans along food chain [53]. In addition, China is the country where more *mcr* positive *S. enterica* strains are identified. This is consistent with the high rates of use of colistin in livestock and veterinary medicine, which leads to the emergence of resistance [10]. Nevertheless, in European countries, such as Italy and Portugal, where colistin is frequently used for therapeutic and metaphylactic purposes in animal husbandry, the reports are emerging [10,41,53]. On the other hand, European countries are more engaged in screening and surveillance activities, which justifies the high number of European reports [14,20,48,54,55]. These studies evidence the wide and ubiquitous spread of *mcr* genes around the world. Although the first report of *mcr-1* only occurred in 2015 from an *E. coli* isolate [9], these genes are also carried by *S. enterica* at least since 2008 [56]. Finally, several *mcr*-carrying *S. enterica* isolates show multidrug resistance profiles, with several genes conferring resistance to tetracyclines, beta-lactams including cephalosporins, quinolones, sulfamethoxazole/trimethoprim and streptomycin, which limits the therapeutic options for treatment of *S. enterica* infections.

The existence of colistin resistance genes embedded into mobile genetic elements, such as plasmids, is a huge concern because they can be horizontally spread across different bacteria. Furthermore, *mcr* genes can be located in plasmids encoding other resistance genes, such as *bla*_CTX-M_, *floR* and/or *qnr*, originating strains resistant to several antibiotic classes, including polymyxins, the majority of beta-lactams, including broad-spectrum cephalosporins and monobactams [48,57,58], amphenicols [51] and quinolones [48,59], respectively. For instance, *mcr-1* and *bla*_CTX-M-1_ genes embedded into plasmid IncHI2 were co-transferred from *S. enterica* isolated from swine retail meat by conjugation under colistin selection [41]. The co-selection of resistance might compromise treatment of complicated gastroenteritis and invasive infections caused by *S. enterica*.

## 6. Conclusion

Here we reviewed the epidemiology of *mcr*-like genes identified in *S. enterica* serovars. It is not expected that colistin will be an antibiotic to treat human enteric fever or gastroenteritis caused by this pathogen; nonetheless, *mcr*-like genes are carried in conjugative plasmids that spread among bacterial populations. The zoonotic feature of *S. enterica* cannot be neglected and plasmid-mediated colistin resistance genes may reach human microbiota through the food chain. Genetic multidrug resistant platforms can be selected not only by colistin but also by the other antibiotics used in livestock, such as quinolones. It is of paramount importance to understand where resistant pathogens are emerging in order to implement infection control measures to prevent their spread. Emergence of *mcr*-like genes are not confined to Asia, as initially supposed, and are found in countries where a higher antibiotic restriction is used in animal production, even in strains isolated ten years ago, raising questions of the stability of these plasmids in bacterial populations, their impact on bacterial fitness. Further research on *mcr*-like genes in zoonotic pathogen populations is necessary to unveil the true impact in human health and to manage colistin use to minimize selection, proliferation and spread of drug-resistant bacteria.

## Figures and Tables

**Table 1:** Reports of *mcr*-like genes identified in *Salmonella enterica*.

Organism Identified	Source of Isolates	Geographical Distribution	Date of Isolation	Identified Gene/Variant	Key Points/Conclusions	Reference
5 *S*. Typhimurium	Isolates from sick swine, duck and chicken from farms	China	2007–2015	*mcr-1*	The high rate of colistin resistance and low *mcr-1* positive rates showed that the plasmid-mediated colistin resistance was not the main mechanism conferring colistin resistance among *Salmonella* isolates	[60]
3 *S*. Typhimurium 1 *S*. Rissen	Swine faeces and swine lymph node	Spain	2009–2011	*mcr-1*	First report of *mcr-1* in *Salmonella* strains Hypothesis: worldwide distribution of this plasmidic element	[46]
4 *S*. Typhimurium	Swine, poultry and cattle food products	Portugal	2011–2012	*mcr-1*	The *mcr-1* gene was already present beyond Asian frontiers in 2011 Plasmid-mediated colistin resistance might be more frequent in Europe than initially thought	[41]
4 *S*. Typhimurium 1 *S*. Derby 1 *S*. Indiana 1 *S*. London	Retail chicken and pork Eggs Retail frozen dumpling	China	2011–2016	*mcr-1*	There is a trend for *Salmonella* spp. becoming a reservoir for the *mcr-1* gene The *mcr-1* gene was already present in *Salmonella* spp. isolates in China in 2011	[61]
14 *S*. Typhimurium 3 *S*. Anatum 1 *S*. Albany 1 *S*. Newport	Human clinical sources; sick food producing animals (pigs and chickens)	Taiwan	2012–2015	*mcr-1*	*mcr-1* gene was carried on distinct plasmids *mcr-1* may have been widespread and become prevalent in zoonotic pathogens in this country	[62]
25 *S*. Typhimurium 3 *S*. Enteritidis	Human clinical sources	China	2012–2015	*mcr-1*	Specific genetic background is required for acquisition and maintenance of *mcr-1* bearing mobile elements Insertion of a *mcr-1* carrying mobile element into the backbone of plasmid might be responsible for one of the modes of *mcr-1* transmission in *Salmonella*	[63]
8 *S*. Typhimurium 1 *S*. Paratyphi B *var* Java 1 *Salmonella* Virchow	Human faeces	UK	2012–2015	*mcr-1*	Several *Salmonella* Typhimurium isolates associated with travel to South-East Asia First report of identification of *mcr-1* in the UK	[64]
2 *S*. Paratyphi B *var* Java phage type Colindale	Poultry meat	Imported from Europe				
19 *S*. Typhimurium 1 *S*. London 1 *S*. Heidelberg	Cecum samples from pig at slaughter	China	2013–2014	*mcr-1*	Horizontal transfer of *mcr-1* harbouring plasmids might have also contributed to spread of *mcr-1* in *Salmonella* spp. Other drug-resistance genes were always co-transferred with *mcr-1*	[51]
21 *S*. Typhimurium 5 *S*. Newport	Food producing animals (chicken, pig seafood, beef)	China	2013–2015	*mcr-1*	Hypothesis that *mcr-1* bearing plasmids might have strong association with specific serotypes of *Salmonella*	[65]
1 *S*. Typhimurium	Ready to eat pork products	China	2014	*mcr-1*	Importance of the role played by *Salmonella* Typhimurium in the dissemination of MDR genes First report on the epidemiological prevalence and detection of *Salmonella* and *mcr-1* gene among ready to eat pork samples in China	[66]
4 *S*. Typhimurium	Human clinical sources	Denmark	2014–2015	*mcr-1*	*mcr-1* producing isolates in patients with travel history to Asia *mcr-1* producing isolates in patients with no travel history is worrying as the spread of *mcr-1* could in the future be present in foodborne outbreaks with *Salmonella* or *E. coli*	[67]
3 *S*. Typhimurium	Human clinical sources (stool and urine)	Colombia	2015–2016	*mcr-1*	Three common resistance genes were identified in the *Salmonella* Typhimurium isolates, including *bla*_TEM-1_, *qnrB19*, and *tet(B)* Transposition of *mcr-1* is the mechanism of mobilization among strains with different genetic backgrounds	[59]
1 *S*. Typhimurium	Retail frozen pork	Brazil	2016	*mcr-1*	First report of *mcr-1* in *Salmonella* Typhimurium in Brazil, highlighting the intercontinental spread of this gene	[68]
3 *S*. Typhimurium	Diarrheal faeces of 3 children (8 months and 15 years old)	China	2016	*mcr-1*	*mcr-1* positive strains were resistant to colistin as well as to third/fourth-generation cephalosporins and sulfamethoxazole/trimethoprim The spread of this *Salmonella* typhimurium clone would pose a great threat to the prevention and control of clinical infections	[69]
1 *S*. Typhimurium *var* Copenhagen	Intestines of pig	Great Britain	No data	*mcr-1*	Plasmid similar to that originally reported in China Dissemination within different *Salmonella* serovars hypothesis Supports the concept of global distribution within a variety of plasmids	[70]
9 *S*. 1,4,[5],12:i:- 2 *S*. Rissen	Human clinical sources (*n* = 4) and pork products (*n* = 7)	Portugal	2011–2015	*mcr-1*	Evidence of the acquisition of *mcr-1* carrying plasmids by two clinically relevant MDR and copper-tolerant clones	[54]
1 *S*. 1,4,[5],12:i:- 1 *S*. Derbi 1 *S. Schwarzengrund* 1 *S*. Paratyphi B	Swine and chicken food products; boot swabs from broiler farm	France	2012–2013	*mcr-1*	These findings reinforce the need to reconsider the use of in-feed colistin in veterinary medicine at a worldwide level	[71]
17 *S*. 1,4,[5],12:i:- 3 *S*. Derby 2 *S*. Bovismorbificans 1 *S*. Newport 1 *S*. Saint Paul 1 *S. Schwarzengrund*	Human clinical sources (*n* = 10), poultry and swine animals (*n* = 2 and 9) and pork food products (*n* = 4)	Italy	2012–2015	*mcr-1*	Italy is one of the main colistin users of European countries and these data are suggestive of gene flow from pigs to humans along the food chain	[53]
1 *S*. 4,[5],12:i:-	Human blood sample	Switzerland	2017	*mcr-1*	The first report of *mcr-1* harbouring *Salmonella enterica* in Switzerland	[72]
1 *S*. Dublin	Pig	France	2002–2014	*mcr-1*	*mcr-1* was present in chickens and pigs at slaughter at least since 2008 in Europe The high diversity among *mcr-1* positive isolates suggested a horizontal transfer	[73]
1 *S*. (4,12:Iv:-)	Chicken	Germany				
1 *S*. Paratyphi B (dTa+)	Chicken skin	Germany	2008	*mcr-1*	Acquisition of the *mcr-1* gene in 2008	[56]
11 *S*. Java	Chicken meat	The Netherlands	2010–2015	*mcr-1*	First finding of a chromosomally located *mcr-1* gene in *E. coli* isolates Ability of *mcr-1* to translocate to the chromosome hypothesis	[74]
1 *S*. Anatum 1 *S*. Schwarzengrund	Turkey meat	Imported meat (no data for origin)				
1 *S. enterica* serovar Indiana	Poultry slaughterhouse (chicken carcasse)	China	2012	*mcr-1*	First report of the complete nucleotide sequence of one *mcr-1* carrying *S*. Indiana strain The strain carried 4 plasmids*,* 1 encoded *bla*_CTX-M-65_ gene along with 20 additional antimicrobial resistance genes	[75]
2 *S*. Schwarzengrund	Poultry meat cuts	Brazil	2013–2016	*mcr-1*	First report of *mcr-1* harbouring *Salmonella enterica serovar* Schwarzengrund Assessment of commercial poultry meat as reservoir of colistin-resistant *Salmonella*	[76]
4 *S. enterica*, 1 belonging to serovar Albany	Intestinal content of diseased chickens	China	2014–2015	*mcr-1*	First report of co-occurrence of *mcr-1* and *bla*_CTX-M-55_ on a single plasmid in *Salmonella enterica* Genetic environment of the *mcr-1* gene is more mobile than expected The selection pressure on the *mcr-1* gene may select for broad-spectrum cephalosporin resistance	[57]
22 *S. enterica*, most of them belong to Albany, Derby, Newport, Mbandaka and Stanley serotypes	Chicken and pig swabs	China	2015–2016	*mcr-1*	Pigs and chickens may be identified as potential sources of *Salmonella* for humans *Salmonella* isolates from food-producing animals frequently exhibited MDR patterns and antimicrobial resistance genes *bla*_CTX-M_, *mcr-1*, and *rmtB* were prevalent	[77]
1 *S*. Typhimurium 1 *S*. Derby 1 *S*. Autoagglutinable	Poultry and pork carcasses	Belgium	2012–2015	*mcr-1* *mcr-2*	First report of detection of *mcr-1* in *Salmonella* isolated from the food chain in Belgium First report of the presence of *mcr-2* in *Salmonella* species isolated from retail meat The *mcr-2* gene seems less transferable and is confined to Belgium	[47]
3 *S*. Typhimurium 7 *S*. monophasic variants of Typhimurium (4,[5],12:i:- and 4,12:i:-)	Human clinical sources	Denmark	2009–2017	*mcr-1* *mcr-3*	One *Salmonella* isolate harbouring both *mcr-1* and *mcr-3* genes (rare combination) Patients with travel history to Asia In addition to *mcr-3*, all strains were found positive for *bla_TEM-1_, strA, strB, sul2* and *tet(A)* or *tet(B),* and most strains were positive for *bla*_CTX-M-55_ and *qnrS*	[48]
4 *S*. Infantis	Broiler meat and broiler chicken	Italy	2016–2017	*mcr-1.1*	First report of the isolation and characterization of four MDR *S*. Infantis, two of them ESBL producers	[58]
1 *S*. Typhimurium 1 *S*. Newport 1 *S*. Blockley	Caecal samples from turkeys	Italy	2014–2015	*mcr-1.1* *mcr-1.2*	Data supports the hypothesis of transmission of *mcr*-positive plasmids between different bacterial species, with the possibility of transmission from animals to humans, or vice versa First report of new *mcr-1* variant in *E. coli* (*mcr-1.13*)	[78]
1 *S*. Typhimurium	Human rectal swab	China	2014	*mcr-1.6*	Identification of a new *mcr-1* gene variant, named *mcr-1.6* Isolated from a healthy human	[79]
1 *S*. 4,[5],12:i:-	Human stool	Canada	2013	*mcr-3.2*	MDR isolate Patient with travel history to Asia (Thailand) Identification of a *mcr-3* variant named *mcr-3.2*	[80]
1 *S*. Typhimurium	Caecal content of a pig at slaughter	Italy	2013	*mcr-4*	Identification of novel plasmid-mediated colistin resistance *mcr-4* gene in *Salmonella* These findings suggest considerable dissemination of the novel gene in Europe	[14]
2 *S*. Typhimurium	Faecal samples of two patients with gastroenteritis	Italy	2016	*mcr-4.2*	First report of *mcr-4* positive bacterial isolates of human origin *Salmonella* species could represent a hidden reservoir for *mcr* genes	[55]
1 *S*. Kedougou	Pig carcass	Spain	2016	*mcr-4.6*	First report of *mcr-4.6*, *a* new *mcr-4* gene variant Development of a multiplex PCR protocol with 100% of specificity and sensibility for five *mcr* genes (1 to 5) for surveillance purposes Detection of two *pmrA/pmrB* point mutations in one colistin-resistant isolate	[20]
2 *S*. 4,[5],12:i:-	Pig and calf carcasses	France		*mcr-1* *mcr-4.2*		
14 *S*. Paratyphi B (dTa+)	Poultry	Germany	2011–2013	*mcr-5*	First report of the *mcr-5* gene The transfer of colistin-resistance-mediating phosphoethanolamine transferase genes from bacterial chromosomes to mobile genetic elements has occurred in multiple independent events raising concern regarding their variety	[15]

MDR, multidrug resistant
